# The periodontal pathogen *Porphyromonas gingivalis* changes the gene expression in vascular smooth muscle cells involving the TGFbeta/Notch signalling pathway and increased cell proliferation

**DOI:** 10.1186/1471-2164-14-770

**Published:** 2013-11-09

**Authors:** Boxi Zhang, Ali Ateia Elmabsout, Hazem Khalaf, Vladimir T Basic, Kartheyaene Jayaprakash, Robert Kruse, Torbjörn Bengtsson, Allan Sirsjö

**Affiliations:** 1 Department of Clinical Medicine, School of Health and Medical Sciences, Örebro University, Örebro, Sweden

**Keywords:** *Porphyromonas gingivalis*, Aortic smooth muscle cells, Proliferation, Gene expression profiling

## Abstract

**Background:**

*Porphyromonas gingivalis* is a gram-negative bacterium that causes destructive chronic periodontitis. In addition, this bacterium is also involved in the development of cardiovascular disease. The aim of this study was to investigate the effects of *P. gingivalis* infection on gene and protein expression in human aortic smooth muscle cells (AoSMCs) and its relation to cellular function.

**Results:**

AoSMCs were exposed to viable *P. gingivalis* for 24 h, whereafter confocal fluorescence microscopy was used to study *P. gingivalis* invasion of AoSMCs. AoSMCs proliferation was evaluated by neutral red assay. Human genome microarray, western blot and ELISA were used to investigate how *P. gingivalis* changes the gene and protein expression of AoSMCs. We found that viable *P. gingivalis* invades AoSMCs, disrupts stress fiber structures and significantly increases cell proliferation. Microarray results showed that, a total of 982 genes were identified as differentially expressed with the threshold log2 fold change > |1| (adjust p-value <0.05). Using bioinformatic data mining, we demonstrated that up-regulated genes are enriched in gene ontology function of positive control of cell proliferation and down-regulated genes are enriched in the function of negative control of cell proliferation. The results from pathway analysis revealed that all the genes belonging to these two categories induced by *P. gingivalis* were enriched in 25 pathways, including genes of Notch and TGF-beta pathways.

**Conclusions:**

This study demonstrates that *P. gingivalis* is able to invade AoSMCs and stimulate their proliferation. The activation of TGF-beta and Notch signaling pathways may be involved in the bacteria-mediated proliferation of AoSMCs. These findings further support the association between periodontitis and cardiovascular diseases.

## Background

*Porphyromonas gingivalis*, a gram-negative asaccharolytic bacterium, has been recognized as a key causative microbe in the pathogenesis of destructive chronic periodontitis. In addition, *P. gingivalis* is able to gain access into the bloodstream and attach to the vascular wall [1]. A great number of epidemiological studies indicate that there is an association between *P. gingivalis* infection and cardiovascular disease [2,3] and DNA of *P. gingivalis* has been detected in coronary stenotic artery plaques of myocardial infarction patients [4]. Furthermore, many in vitro and animal experiments support the connection between *P. gingivalis* infection and the pathogenesis of atherosclerosis. We have previously reported that *P. gingivalis* induces neutrophil ROS-production, sensitizes platelet for epinephrine, down-regulates immune response of T-cells and converts LDL to an atherogenic form [5-8]. Although, it is not easy to conduct systematic studies in human subjects, in vitro studies have shown that *P. gingivalis* can invade different types of human vascular cells, including umbilical vein endothelial cells (HUVECs) [9], coronary artery smooth muscle cells [10], and aortic smooth muscle cells [11].

Virulence factors of *P. gingivalis*, such as lipopolysaccharides (LPS), fimbriae, toxic products of metabolism and proteases have been identified to activate defensive response processes of host cells, leading to release of inflammatory mediators and chronic inflammation [12]. During the last decades, inflammation has been attributed as the key factor beneath atherosclerosis which was formerly considered as a bland lipid storage disease [13,14]. The development of atherosclerosis is due to a complex interaction between multiple risk factors including hypertension, high plasma levels of inflammatory mediators, and hypercholesterolemia [15]. It is possible that *P. gingivalis*, directly or indirectly, induces and supports inflammatory processes in the vessel wall. In atherogenesis, different cell types, including macrophages, monocytes, platelets, endothelial cells, and smooth muscle cells (SMCs), are involved [16].

Vascular smooth muscle cells (VSMCs) are one of the fundamental components of the vessel wall and are involved in atherogenesis, plaque progression and rupture [17]. During atherogenesis, the VSMCs undergo phenotypic modulation from a quiescent to a synthetic state that is activated by various mediators, such as platelet-derived growth factor, and migrate from the media into the intima. In the intima, VSMCs enhance synthesis of extracellular matrix (ECM) and proliferate under the stimulation by specific cytokines. The proliferation of VSMCs and the production of ECM contribute to the plaque growth and the development of fibrotic cap. Furthermore, the production of ECM facilitates accumulation of modified low-density lipoproteins (LDL) through binding to proteoglycans. After binding to proteoglycan, LDL is oxidized, which further activates VSMCs to produce more sulfated proteoglycans and arrest more LDL [18].

Up to now, no study has been conducted to elucidate the possible signaling mechanisms involved in VSMCs challenge with live *P. gingivalis*. The aim of this study is to investigate effects of *P. gingivalis* infection on gene and protein expression and its relation to cellular function of human aortic smooth muscle cells in order to clarify the association between periodontitis and cardiovascular disease.

## Results

### *P. gingivalis* invades AoSMCs

**Figure 1:**
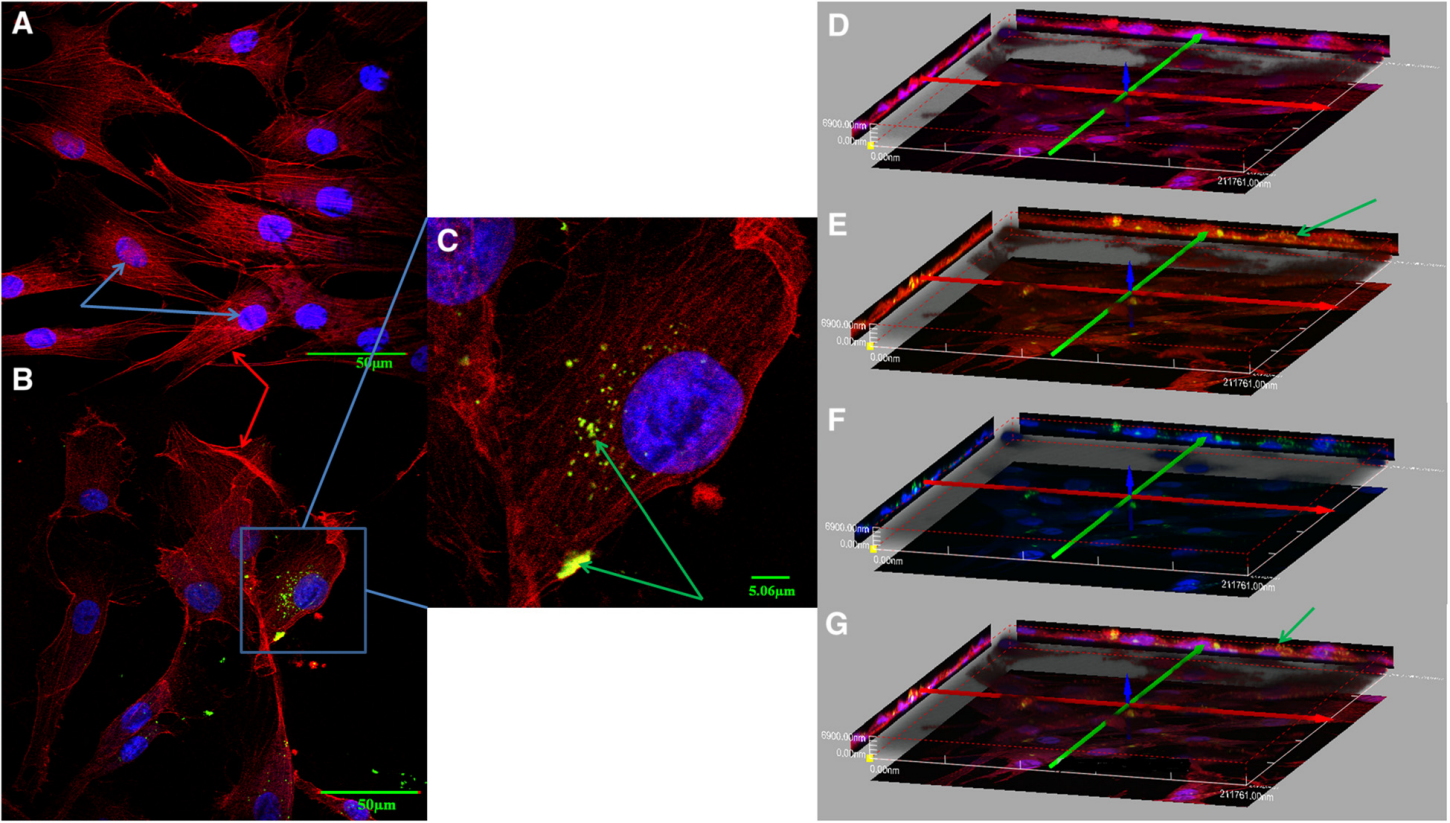
***P. gingivalis *invades AoSMCs and changes the F-actin distribution.** AoSMCs were cultured on glass coverslips in multiwall plates stimulated with **(B and C)** or without **(A)** FITC-stained *P. gingivalis* (green arrows) for 24 h and then stained for F-actin (red arrows) and nucleus (blue arrows). The cells were mounted upside-down and visualized by scanning confocal laser microscopy. The 3D images **(D-G)** are shown by different combination of stained components: F-actin and nucleus **(D)**; F-actin and FITC-stained *P. gingivalis***(E)**; nucleus and FITC-stained *P. gingivalis***(F)**; F-actin, nucleus and FITC-stained *P. gingivalis***(G)**.

Using confocal fluorescence microscopy, we found that *P. gingivalis* at the MOI of 10 invaded AoSMCs and after 24 hours showed mainly at perinuclear localization (Figure 1C). Compared with control samples (Figure 1A), AoSMCs infected by *P. gingivalis* demonstrated a disruption of stress fibers and a relocalization of F-actin to the cell periphery (Figure 1B). The 3D images (Figure 1D-G) show that *P. gingivalis* is able to invade AoSMCs.

### *P. gingivalis* increase AoSMCs proliferation

**Figure 2:**
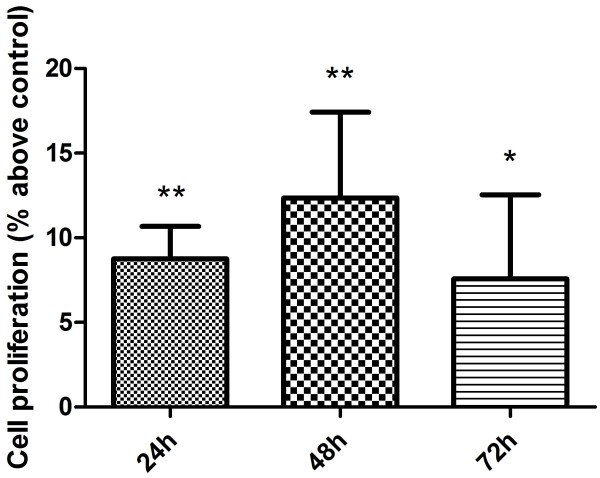
***P. gingivalis *increases AoSMCs proliferation.** The neutral red assay was used to analyze changes in AoSMCs growth after challenge with *P. gingivalis* at the concentration of 10MOI for 24 h in the absence or presence of viable *P. gingivalis*. The results are presented as the mean ± SEM of four separate experiments. *, p < 0.05. **, p < 0.005. n = 4.

The proliferative effects of viable *P. gingivalis* on AoSMCs were examined with the neutral red assay. We found that AoSMCs, after challenging with *P. gingivalis* for 24 h significantly increased the subsequent proliferation in 0.5% serum after 24 h, 48 h, and 72 h, compared with the unstimulated cells, with a maximal effect after 48 h (Figure 2).

### *P. gingivalis* changes gene expression in AoSMCs

A difference in the expression of specific genes in AoSMCs induced by *P. gingivalis*, was identified by setting the threshold of log fold change (logFC) above |1| with adjust p- value (Benjamini-Hochberg) < 0.05. Through analyzing mRNA originated from 4 independent experiments, a total of 982 genes were identified as differentially expressed, in which 438 genes were up-regulated and 544 genes were down-regulated, compared to the uninfected control group. A whole list of differentially expressed genes is provided in supplemental material (Additional file 1: Table S1). The whole data were visualized by CIRCOS [19] (Additional file 2: Figure S1).

**Figure 3:**
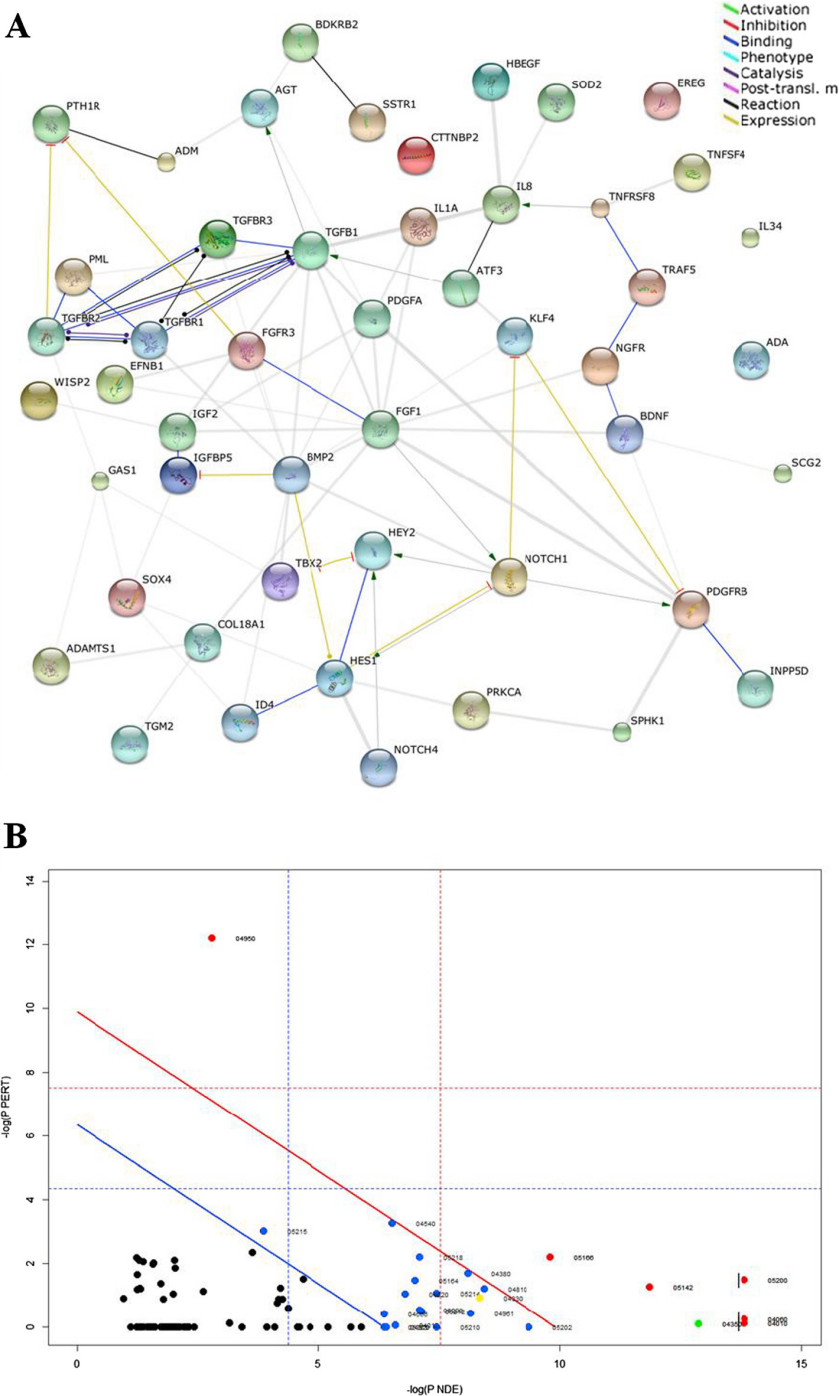
**The protein-protein interaction network and SPIA analysis of the 28 up-regulated genes related to positive regulation of cell proliferation and 21 down- regulated genes related to negative control of cell proliferation.** The network **(A)** was mapped using STRING, and the confidence parameter was set as 0.15. Lines between proteins stand for possible interactions, and are color-coded based on the type of interaction. In SPIA analysis **(B)**, X-axis indicates probability to observe differentially expressed genes on the pathway; y-axis refers to the probability to observe perturbation of genes within pathways. Each number refers to a KEGG pathway ID. Pathways above the blue line are significant at 5% after FDR correction; those above the red lines are significant at 5% after Bonferroni correction. 49 genes were analyzed by SPIA analysis. The Notch pathway is shown as a yellow dot and TGF-beta pathway is shown as a green dot.

For the differentially expressed genes, we found that 28 up-regulated genes (Additional file 3: Table S2) were significantly related to the GO term of positive regulation of cell proliferation (GO: 0008284, p-value < 1.6E-6); 21 down-regulated genes (Additional file 4: Table S3) were related to the GO term of negative regulation of cell proliferation (GO: 0008285, p-value < 2.5E-3). The functional interaction network for the genes involved in these two categories is shown in (Figure 3A).

### Pathway analysis of genes regulated by *P. gingivalis* using SPIA

The 28 up-regulated genes related to the GO term of positive regulation of cell proliferation and 21 down-regulated genes related to the GO term of negative regulation of cell proliferation were inserted in the R platform and analyzed using SPIA package. Significant pathways were picked out by combining the fold change of the genes and pathways topology information. For each pathway, a novel parameter, termed perturbation, was measured by the position of specific genes within the 134 KEGG pathways. Genes with a high hierarchical position would have more power to determine whether the genes are enriched in a certain pathway. For a total of 49 genes, SPIA analysis showed that these genes were enriched in 25 pathways (FDR < 0.05), in which, 7 pathways were considered significant with a level of 5% after Bonferroni correction (Figure 3B). All 25 pathways are listed in supplemental material (Additional file 5: Table S4).

### Validation of microarray data in Notch and TGF-beta pathway

**Figure 4:**
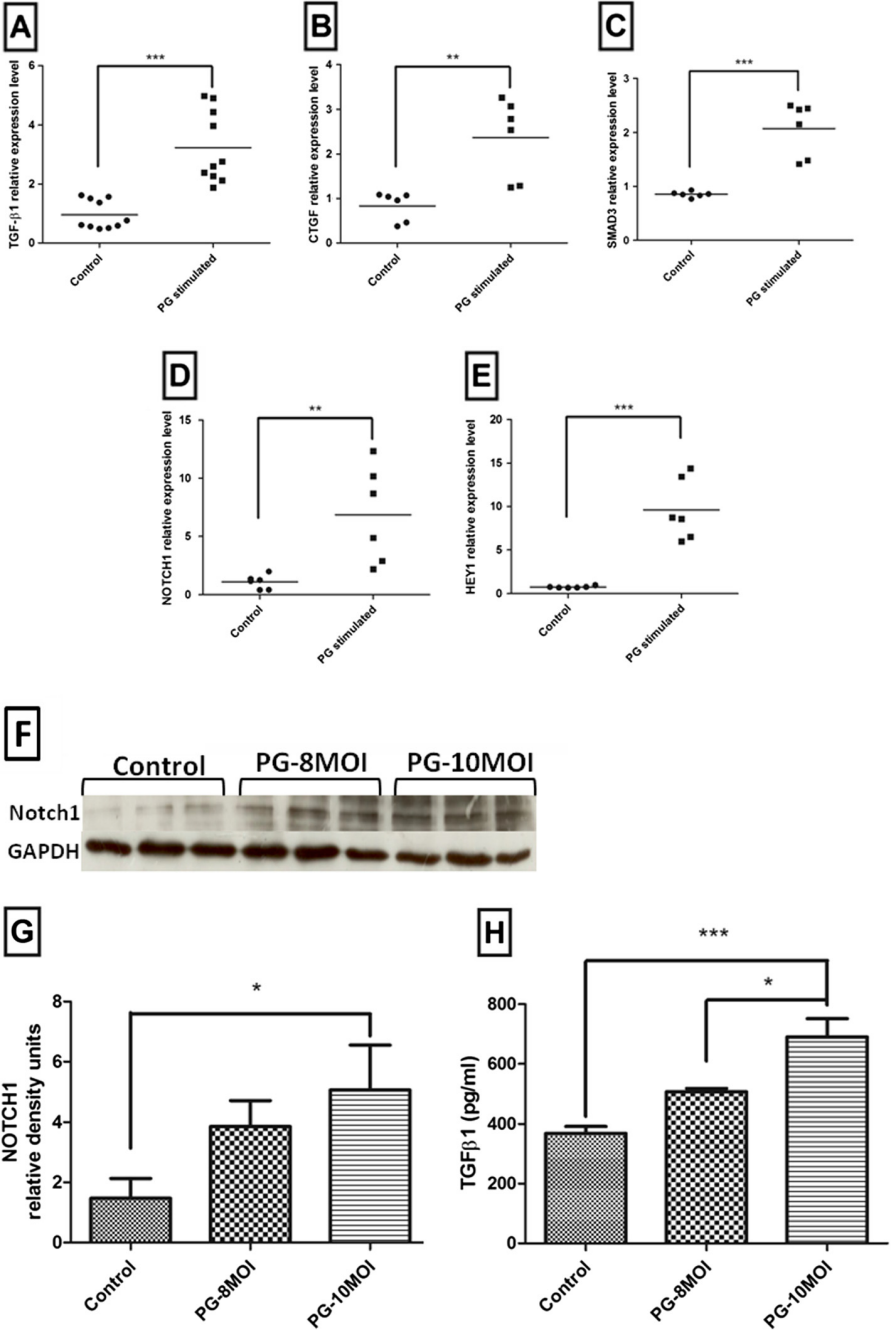
**The expression of selected genes and proteins in AoSMCs stimulated with *P. gingivalis*.** Quantitative real-time PCR results demonstrate relative transcription for TGF-β1 **(A)**, CTGF **(B)**, SMAD3 **(C)**, NOTCH1 **(D)**, and HEY1 **(E)** of AoSMCs stimulated (*P. g*) or unstimulated (control) with *P. gingivalis* at 10 MOI for 24 h. All these results were normalized by the gene expression level of the house-keeping gene GAPDH. Representative western blot showing NOTCH1 protein expression levels of AoSMCs exposed to *P. gingivalis* at 8 and 10 MOI **(F)**. Quantification of NOTCH1 protein expression levels by densitometry is shown in **(G)**. NOTCH1 density signals were normalized to GAPDH signal values. *P. gingivalis* resulted in a dose-dependent (MOI 8 and 10) induction of TGF-β1 protein expression **(H)**. *, p < 0.05; **, p < 0.005; ***, p < 0.0001. **A**, n = 10; **B**-**E**, n = 6; **F**, **G**, n = 3; **H**, n = 3-4.

In this study, we focused on TGF-beta and Notch pathway that are known to be involved in the proliferation of AoSMCs. Several differentially expressed genes in these two pathways were picked out and validated by qPCR. The gene TGF- β1, which belong to the TGF-beta pathway, showed up-regulation by *P. gingivalis* in both the microarray and the qRT-PCR assay (Figure 4A), as well as in protein level (Figure 4H). We further validated the gene of connective tissue growth factor (CTGF), which has been indicated to cooperate with TGF-beta to induce fibrosis [20]. The qPCR analysis of the CTGF gene (Figure 4B) confirms the *P. gingivalis*-mediated up-regulation of this gene and complements the results from the microarray. The third gene selected for validation of microarray data was SMAD family member 3 (SMAD3) (Figure 4C), which has previously been shown to be a signaling components of the TGF-beta superfamily [21]. In Notch pathway, we focused on two genes, Notch1 (Figure 4D) and Hairy/enhancer-of-split related with YRPW motif protein 1 (HEY1) (Figure 4E). Notch1, which functions as a membrane-bound signaling molecule and takes part in various defense and immune responses, showed an up- regulation in *P. gingivalis*-infected AoSMCs in both the microarray and qPCR results. This increase in gene expression was associated with an elevation in protein level, using western blot (Figure 4G and 4F). HEY1 is a downstream gene of Notch1 in the Notch pathway. We found that *P. gingivalis* increased the expression of this gene in AoSMCs more than 10 fold, both in the qPCR and the microarray.

## Discussion

Many risk factors have been identified to contribute to the development of atherosclerosis and cardiovascular disease. Classical risk factors include high circulating levels of LDL, smoking, and low physical activity. However, up till 50% of patients with cardiovascular disease do not possess any of the classical risk factors [22]. It is believed that the immune system participates in the development of atherosclerosis and inflammation and infection have been considered as key factors [23]. Increasing evidence has implicated that specific microorganisms, including the periodontal pathogen *P. gingivalis*, are involved in the progression of atherosclerosis. In this study, we focused on the interaction between *P. gingivalis* and vascular smooth muscle cells. We found, by using confocal microscopy 3D analysis, that *P. gingivalis* invades AoSMCs, reorganizes the actin cytoskeleton and causes AoSMCs proliferation, the latter considered as a key process in atherosclerosis. Although, proliferative effects of *P. gingivalis* infection of SMCs have previously been reported [24], the mechanisms involved are uncertain. We used a comprehensive bioinformatics analysis and studied the gene expression profiling of smooth muscle cells after challenge with viable *P. gingivalis*, which gave us an insight of the effects of this periodontal bacterium on the vessel wall.

By using microarray analysis, we found that 982 genes were differentially expressed in *P. gingivalis*–infected AoSMCs, compared to uninfected control samples. In order to clarify whether genes contributing to cell proliferation are involved during *P. gingivalis* infection, gene ontology analysis was performed. We found that differentially expressed genes were significantly enriched in the GO categories, including positive regulation of cell proliferation for up-regulated genes and negative regulation of cell proliferation for down-regulated genes. In these two categories, growth factors and their receptors were enriched, such as heparin-binding growth factor 1 (FGF1), platelet-derived growth factor subunit A (PDGFA), fibroblast growth factor receptor 3 (FGFR3) and beta-type platelet- derived growth factor receptor (PDGFRB). Interestingly, we also found a great number of genes belonging to Notch and TGF-beta pathway. The result of SPIA analysis showed that the differentially expressed genes belonging to these two GO categories are enriched in NOTCH and TGF-beta pathway, so as the total up-regulated genes by *P. gingivalis* treatment (Additional file 6: Figure S2). Among the genes that were differently expressed by *P. gingivalis*, GUCY1A3 (36.8 fold) and GUCY1B3 (36.5 fold) are the top 2 up-regulated genes. Both genes are associated with components belong to the downstream of Notch signaling pathway [25]. Furthermore, within Notch signaling pathway, *P. gingivalis* up-regulated three Notch receptors (Notch1, Notch3, Notch4). Notch signaling pathway regulates organogenesis and critical cellular processes such as cardiomyocyte proliferation and differentiation during heart development [26]. Notch1 has been shown to play an important role in SMCs proliferation, migration and survival. Neointimal formation in Notch1 general heterozygous knockout (Notch1 +/-) mice was remarkably suppressed compared to wild type mice [27]. Indeed, Notch signaling also plays important role in the pathogenesis of common vascular proliferative syndromes including atherosclerosis and restenosis [28]. In addition, we found that the bHLH genes of the Hes/Hey families also were highly induced by *P. gingivalis*, including HES1 (35.0 fold), HES4 (5.8 fold), HES5 (23.5 fold), HEYL (29.6 fold), HEY1 (35.0 fold), and HEY2 (26.1 fold). Hes/Hey familiy is identified as the target genes of various Notch receptors [29,30]. In correlation, lipopolysaccharide from *P. gingivalis* has been shown to activate Notch1 signaling and induce the production of HES1 and HEY1 [31]. Other target genes like JAG1, SDC2 and SNAI2 were also demonstrated to be up-regulatied [32-34]. All these results complement to the SPIA analysis, further demonstrating that the Notch pathway is significantly activated in AoSMCs in response to *P. gingivalis*.

**Figure 5:**
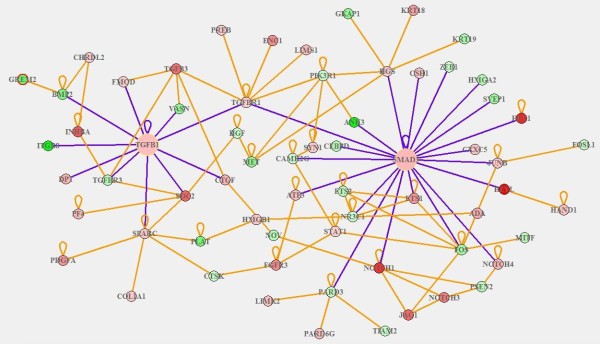
**The graph of gene-gene association among TGF-β1, Smad3 and other differentially expressed genes in AoSMCs exposed to *P. gingivalis*.** Each dot refers to one gene and the color of dots refers to the log fold change of the gene expression level. Red dots indicate up-regulated genes. Green dots indicate down-regulated genes. The deeper is the color, the bigger fold change of the genes.

**Figure 6:**
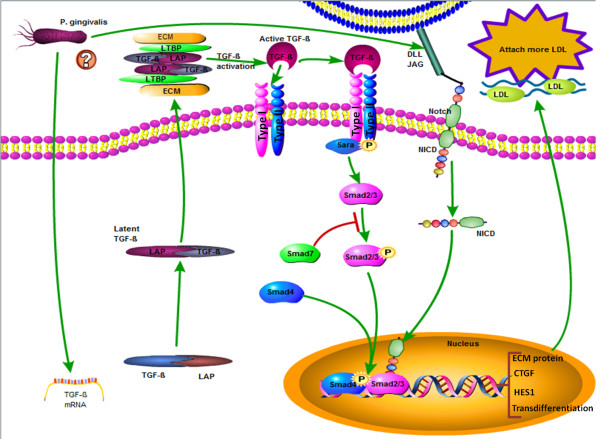
**TGF-β and NOTCH activation in AoSMCs stimulated with *P. gingivalis*.** Treatment of AoSMCs with viable *P. gingivalis*, increases the mRNA expression level of TGF-beta. TGF-beta is secreted as a latent complex which can be activated by a number of agents. The activation of TGF-beta interacts with TGFRII and this complex further phosphorylates TGFRI to activate the Smad pathway by phosphorylation at the carboxyl terminus. During this process, Smad anchor for receptor activation (SARA) can facilitate TGF-beta receptor complex to bind to R-Smads (Smad 2/3). Once the R-Smads being phosphorylated, they bind to Co-Smads to form a complex which go into nucleus and regulates the transcription of several targets genes involved in atherosclerosis. Beside this, TGF-beta and NOTCH pathway can collaborate with each other by direct interaction between Smad3 and NICD.

We noticed that the TGF-beta pathway was also significantly activated in AoSMCs by *P. gingivalis*. TGF-beta can cooperate with Notch pathway in the regulation of SMCs differentiation [35]. Although the growth of normal human SMCs is inhibited by TGF-beta, the growth of cells isolated from human atherosclerotic lesions is markedly elevated by TGF-beta pathway activation, accompanied by an increase in collagen synthesis [36]. In consent, previous reports have revealed in vivo, using balloon injury models, that increased levels of TGF-β1 signaling enhance the intimal thickness and induce SMCs proliferation and differentiation [37]. Through the activation of TGF-beta, the glycosaminoglycan synthetic machinery of AoSMCs can be modulated and bind more LDL [38]. We also found that the gene of Smad3 is highly induced by *P. gingivalis* and when Smad3 levels are elevated, TGF-beta stimulates SMCs to proliferate and accelerate neointimal formation [39,40]. In order to understand the association between TGF-β1 and Smad3 and how they interact with other differentially expressed genes, we have visualized gene-gene interactions by GeneAnswers package (Figure 5). We found that there is a direct connection between TGF- β1 and Smad3 through the TGF receptor type I (TGFRI). Activation of the TGF-beta pathway leads to binding of TGF-beta to TGF receptor type II (TGFRII), and then, this complex binds to TGFRI, resulting in TGFRI phosphorylation [41] and activation of the downstream Smad pathway. The Smad pathway regulates the transcription of several target genes [42], such as CTGF and Elastin (ELN) (Figure 6). The results from GeneAnswers package also showed there is a crosstalk between smad3 and Notch1. This connection is due to the direct protein–protein interaction between Notch intracellular domain (NICD) and Smad3 [43]. Due to the fact that the TGF- beta and Smad3 are over-expressed after arterial injury, as well as the activation of Notch pathway, we suggest that these signaling mechanisms are involved in *P. gingivalis*- induced differentiation and proliferation of AoSMCs.

## Conclusions

In summary, this study suggests that the periodontal pathogen *P. gingivalis* stimulates AoSMCs proliferation through activation of the TGF-beta and Notch signaling pathways and thus enhances the progression of atherosclerosis, which further supports an association between periodontitis and cardiovascular disease.

## Methods

### Culture of SMCs

Human primary AoSMCs (Invitrogen, Stockholm, Sweden) were cultured in 231 smooth muscle cell culture medium (Gibco, Carlsbad, CA) containing recommended supplements. The cells were cultured in 75 cm [2] explants culture flasks (TPP, Trasadingen, Switzerland) and placed in cell culture incubator at 37°C with 5% CO_2_ and 95% air. Cells were subcultured after confluence. Cells from passage 5-10 were used in this study.

### Porphyromonas gingivalis

The *P. gingivalis* ATCC 33277 (American Type Culture Collection, Manassas, VA) were cultured in fastidious anaerobe broth (29.7 g/liter, pH 7.2) in an anaerobic chamber (80% N_2_, 10% CO_2_, and 10% H_2_, 37°C) (Concept 400 Anaerobic Workstation; Ruskinn Technology Ltd., Leeds, United Kingdom). The bacteria were harvested after 3 to 4 days by centrifugation for 10 min at 10000 rpm, followed by washing and resuspension in Krebs-Ringer-Glucose (KRG) buffer. The supernatant was removed from bacteria pellet, which was then washed with KRG buffer supplemented with 1.1 mM CaCl_2_. The concentration of *P. gingivalis* was measured by counting CFU (Colony-forming unit) of different dilutions of bacteria on blood agar after 5 to 7 days. The optical density (OD) at 600 nm of the bacteria suspension was measured with a spectrophotometer (BioPhotometer plus) (Eppendorf AG, Hamburg, Germany) to correlate to the concentration (CFU/ml) of the bacteria.

### Bacterial inoculation

AoSMCs were dissociated using 3 ml trypsin/EDTA solution (Gibco, Carlsbad, CA) and transferred to 12 ml microcentrifuge tube, centrifuged at 14,000 rpm for 4 min, re- suspensed in fresh medium, and seeded at a density of 150,000 cells per well of the plate coated with Type I collagen (Gibco, Carlsbad, CA). Cells were serum starved for 24 hour using DMEM medium (Gibco, Carlsbad, CA) with 0.5% FBS (Sigma, St. Louis, MO), 2 mM L-glutamin (Gibco, Carlsbad, CA) and antibiotics (Gibco, Carlsbad, CA). After 24 hour serum starvation, medium were discarded and AoSMCs washed and resuspended with fresh DMEM medium. The AoSMCs were challenged with viable *P. gingivalis* with the concentration of 8 or 10 MOI for 24 hours.

### Confocal fluorescence microscopy

*P. gingivalis* was incubated with 2 g/ml fluorescein isothiocyanate (FITC) (Sigma, St. Louis, MO), dissolved in carbonate-bicarbonate buffer (pH 9.2), for 1 hour at room temperature with gentle agitation in dark. After wash twice in PBS, the concentration of bacteria was measured by OD at 600 nm. The viability of FITC-labeled *P. gingivalis* was confirmed by viable count analysis. AoSMCs were cultured on type I collagen coated glass cover slips, in 6 well cell culture plates. After serum starvation, cells were challenged with FITC-labeled *P. gingivalis* (10 MOI) for 24 hour, followed by fixation with 4% paraformaldehyde (PFA) for 30 minutes at room temperature. The F-actin of the cells was stained by incubation with Alexa Fluor 594 Phalloidin (Molecular Probes, Eugene, OR) in the dark for 30 minutes. The nucleus was stained using 4’6-diamidino-2-phenylindole (DAPI) (Sigma, St. Louis, MO) for 10 minutes in dark, followed by washing twice with PBS. The cover slips were dried in room air, and then, mounted onto microscope glass slides using mounting medium (Pertex; Histolab Products, Gothenburg, Sweden). A scanning confocal laser microscope, (FluoViewTM FV1000 Confocal Laser Scanning Biological Microscope; Olympus, Hamburg, Germany) was used to visualize the stained cells. The images were captured in 60× objective using oil immersion lens, whereafter the images were processed using FV10-ASW viewer 2.0 software (Olympus, Hamburg, Germany). The 3D images were created by stacking 77 pieces of slices which were captured every 0,1 μm over each other.

### Proliferation assay

In order to investigate the proliferation responses, serum-starved AoSMCs were incubated with viable *P. gingivalis* for 24 h (see above), whereafter the medium was replaced with medium containing 0.5% FBS for 24 h, 48 h and 72 h. The proliferation responses were monitored using the neutral red assay described by Guillermo et al [44]. Briefly, neutral red (Sigma-Aldrich, St. Louis, MO) was dissolved in the cell culture medium at the concentration of 40 μ g/ml and incubated overnight at 37°C. The medium of the samples was aspirated out and cells were washed twice with PBS, whereafter 1 ml of neutral red medium (40 μ g/ml) was added to each well of the plate. After 2  h incubation at 37°C, the neutral red medium was removed. The neutral red was extracted from the cells by adding 1 ml destain solution (50% of 96% ethanol, 49% MQ water, 1% glacial acetic acid), followed by measurements of OD absorbance at 540 nm in a microtiter plate reader. (SpectraMax 340, Microplate Reader; Molecular Devices Corp., Sunnyvale, CA).

### Microarray gene expression analysis

After 24 hour incubation with *P. gingivalis*, cells were harvested and RNA was extracted from the cells according to the protocol of RNeasy Kit (Omega Bio-Tek, Norcross, GA). In order to minimize experimental and technical errors in our array analysis, we produced 4 biological replications and swapped the dyes for two of the arrays. The quality of RNA was evaluated using the Agilent Bioanalyze (Agilent, Santa Clara, CA) and nanodrop 2000 (Thermo, Wilmington, DE). The RNA samples were hybridized and scanned by Agilent Microarray Scanner (Agilent, Santa Clara, CA). Agilent human whole genome 4x44 arrays (Agilent, Santa Clara, CA) and protocols were used for gene expression profiling analysis. For gene expression level investigations, i.e. the analysis of whole- gene variation with genotype, the .txt files obtained from Feature Extraction software (version 6.1.1, Agilent Technologies) was preprocessed using limma package [45] offered by Bioconductor repository [46]. The raw data were normalized and log2- transformed. Linear model was tried to identify differentially expressed genes. Fold change and adjusted p-value (false discovery rate, FDR) was calculated and genes with fold change ≥ 2 and FDR < 0.05 were considered as differentially expressed genes and were used for further analysis in this study. In order to characterize gene functions related to proliferation, up-regulated and down- regulated differentially expressed genes were input into DAVID [47], separately, and the functional interaction networks was built using STRING [48].

### KEGG pathway enrichment analysis by Signaling Pathway Impact Analysis (SPIA)

Signaling Pathway Impact Analysis (SPIA) algorithm [49,50] was applied to find the specific KEGG signaling pathways (database downloaded from KEGG’s website on: 03/21/2012) that included the differential expressed genes related to cell proliferation. SPIA integrates the information from all genes that were considered to be differentially expressed and the vector of log2 fold change of each gene to do the enrichment analysis. 134 pathways at this version are looked through to find significantly (false discovery adjusted global p- value cut-off of 0.1) modulated pathways.

### Quantitative real-time PCR

cDNA were made using High Capacity cDNA Reverse Transcription Kits (PERkin- Elmer Applied Biosystems, Foster City, CA) following the recommended protocol. Quantitative real-time PCR was performed with an ABI Prism 7900 HT Sequence Analyzer using the manufacturer’s recommended protocol (PERkin-Elmer Applied Biosystems, Foster City, CA) to validate differentially expressed genes of interest. The PCR primer and probes (PERkin-Elmer Applied Biosystems, Foster City, CA) for TGF-β1 (Hs00998133_m1), CTGF (Hs01026927_g1), Notch1 (Hs01062014_m1), HEY1 (Hs01114113_m1), and SMAD3 (Hs00969210_m1) were applied in this study and GAPDH was used for normalization.

### Western blot assay

Proteins were extracted from AoSMCs, which had been unstimulated or stimulated for 24 h with viable *P. gingivalis*, using RIPA Buffer (Sigma-Aldrich, St. Louis, MO) containing a protease inhibitor cocktail (Sigma-Aldrich, St. Louis, MO). The total protein concentration was determined using the BCA protein assay kit (Thermo Scientific, Rockford, USA). An equal amount of each sample (20 μg) was electrophoresed on 10% SDS–PAGE and transferred onto nitrocellulose membrane (Bio-Rad, Hercules). After blocking in non-fat dried milk, membranes were probed overnight at 4°C using rabbit polyclonal anti-cleaved Notch1 (Novus Biologicals, Cambridge, UK) in 1: 2,000 dilution. Rabbit polyclonal anti-GAPDH in 1:15,000 (Santa Cruz Biotechnology, Dallas, Texas) dilution was used as loading control. Blots were then incubated with anti-rabbit IgG (Santa Cruz Biotechnology, Dallas, Texas) and visualized using an enhanced chemiluminescence system (GE Healthcare Biosciences, Pittsburgh, PA) and exposed to Hyperfilm enhanced chemiluminescence (GE Healthcare Biosciences, Pittsburgh, PA). Densitometric analysis was performed using NIH software package Image J (ImageJ 1.32j; NIH, Bethesda, MD).

### Enzyme-linked immunosorbent assay (ELISA)

The supernatants from AoSMCs challenged for 24 h with different concentrations of viable *P. gingivalis* were collected and centrifugated at 1500 × g for 5 min at 4°C, whereafter, the supernatants were stored at -80°C until use. ELISA was performed using supernatants to quantify TGF-β1 (BD OptEIA Set Human TGF-β1, BD Biosciences, USA) according to the manufacturer's instructions.

### Statistical analysis

The Benjamini-Hochberg procedure was used to find the differentially expressed genes in microarray data. Student's t-test was used for statistical comparisons of two groups and one-way ANOVA with appropriate post-tests was used for more than two groups, and were performed using Graphpad Prism software.

### Availability of supporting data

All microarray data were deposited into the ArrayExpress database (E-MTAB-1922). Other supporting data are available as additional files.

## Competing interests

The authors declared that they have no competing interests.

## Authors’ contributions

BZ and AAE contributed to the design of the study and carried out the experiments. KJ analyzed the results of the confocal fluorescence microcopy. VTB analyzed the western blot results. BZ and RK carried out the microarray experiments. HK participated in the study design and experiment processes. TB and AS conceived the study, coordinated the research and had the financial responsibility. BZ did the data mining for microarray experiments and wrote the manuscript. All authors read and approved the final manuscript.

## Supplementary Material

Additional file 1: Table S1 List of differentially expressed genes in *P. gingivalis* treated samples. Click here for file

Additional file 2: Figure S1 Visualization of the sequence annotation and relative expression level using CIRCOS. The outer layer shows the chromosomes and the band of chromosomes. The second layer refers to the down-regulated sequences (in green) and the up-regulated sequences (in red). The light lines of the third layer refer to the significantly differentially expressed sequences. The gene names of the significantly down-regulated sequences are shown in blue color and the gene names of the significantly up-regulated sequences are shown in black color. Click here for file

Additional file 3: Table S2 Up-regulated genes in AoSMCs stimulated by *P. gingivalis* with a positive regulation of cell proliferation. Click here for file

Additional file 4: Table S3 Down-regulated genes in AoSMCs stimulated by *P. gingivalis* with a negative regulation of cell proliferation. Click here for file

Additional file 5: Table S4 KEGG pathways for 28 up-regulated genes related to the GO term of positive regulation of cell proliferation and 21 down-regulated genes related to the GO term of negative regulation of cell proliferation analyzed by SPIA (FDR < 0.05). Click here for file

Additional file 6: Figure S2 SPIA analysis on up-regulated genes in AoSMCs exposed to *P. gingivalis*. X-axis indicates probability to observe differentially expressed genes on the pathway; y-axis refers to the probability to observe perturbation of genes within pathways. Each number refers to a KEGG pathway ID. Pathways above the blue line are significant at 5% after FDR correction, those above the red lines are significant at 5% after Bonferroni correction.438 up-regulated genes were analyzed by SPIA analysis. The Notch pathway is shown as a yellow dot and TGF-beta pathway is shown as a green dot. Click here for file
