# Supplementary material for: The periodontal pathogen Porphyromonas gingivalis changes the gene expression in vascular smooth muscle cells involving the TGFbeta/Notch signalling pathway and increased cell proliferation
Source: BMC Genomics. 2013 Nov 9;14:770. doi: 10.1186/1471-2164-14-770 (PMC3827841; doi:10.1186/1471-2164-14-770)
Supplement: Additional file 2: Figure S1 — Visualization of the sequence annotation and relative expression level using CIRCOS. The outer layer shows the chromosomes and the band of chromosomes. The second layer refers to the down-regulated sequences (in green) and the up-regulated sequences (in red). The light lines of the third layer refer to the significantly differentially expressed sequences. The gene names of the significantly down-regulated sequences are shown in blue color and the gene names of the significantly up-regulated sequences are shown in black color. [file 1471-2164-14-770-S2.pdf]

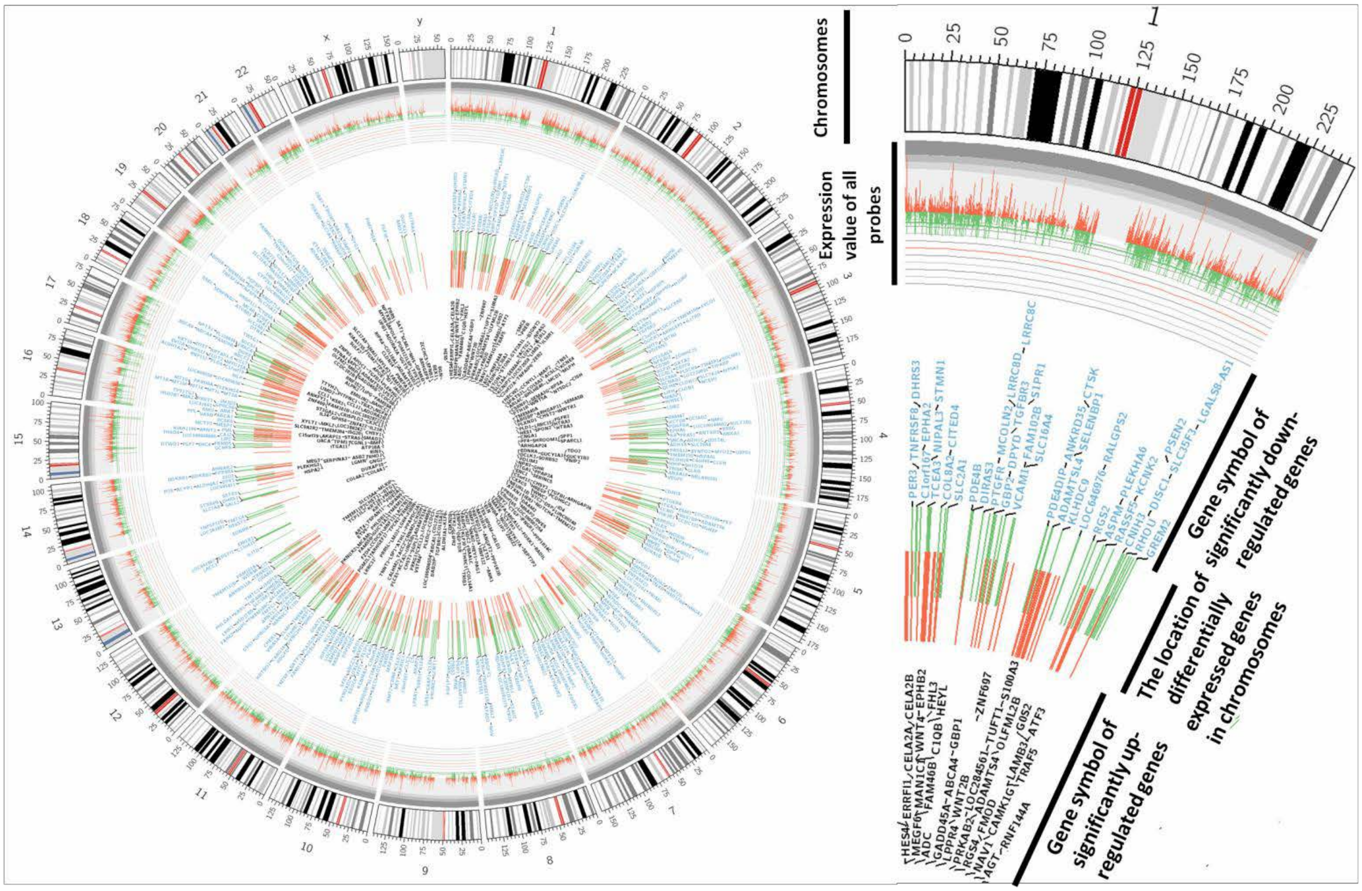

Fig. S1. Visualization of the sequence annotation and relative expression level using CIRCOS. The outer layer shows the chromosomes and the band of chromosomes. The second layer refers to the down-regulated sequences (in green) and the up-regulated sequences (in red). The light lines of the third layer refer to the significantly differentially expressed sequences. The gene names of the significantly down-regulated sequences are shown in blue color and the gene names of the significantly up-regulated sequences are shown in black color.
