# Supplementary material for: The periodontal pathogen Porphyromonas gingivalis changes the gene expression in vascular smooth muscle cells involving the TGFbeta/Notch signalling pathway and increased cell proliferation
Source: BMC Genomics. 2013 Nov 9;14:770. doi: 10.1186/1471-2164-14-770 (PMC3827841; doi:10.1186/1471-2164-14-770)
Supplement: Additional file 6: Figure S2 — SPIA analysis on up-regulated genes in AoSMCs exposed to P. gingivalis. X-axis indicates probability to observe differentially expressed genes on the pathway; y-axis refers to the probability to observe perturbation of genes within pathways. Each number refers to a KEGG pathway ID. Pathways above the blue line are significant at 5% after FDR correction, those above the red lines are significant at 5% after Bonferroni correction.438 up-regulated genes were analyzed by SPIA analysis. The Notch pathway is shown as a yellow dot and TGF-beta pathway is shown as a green dot. [file 1471-2164-14-770-S6.pdf]

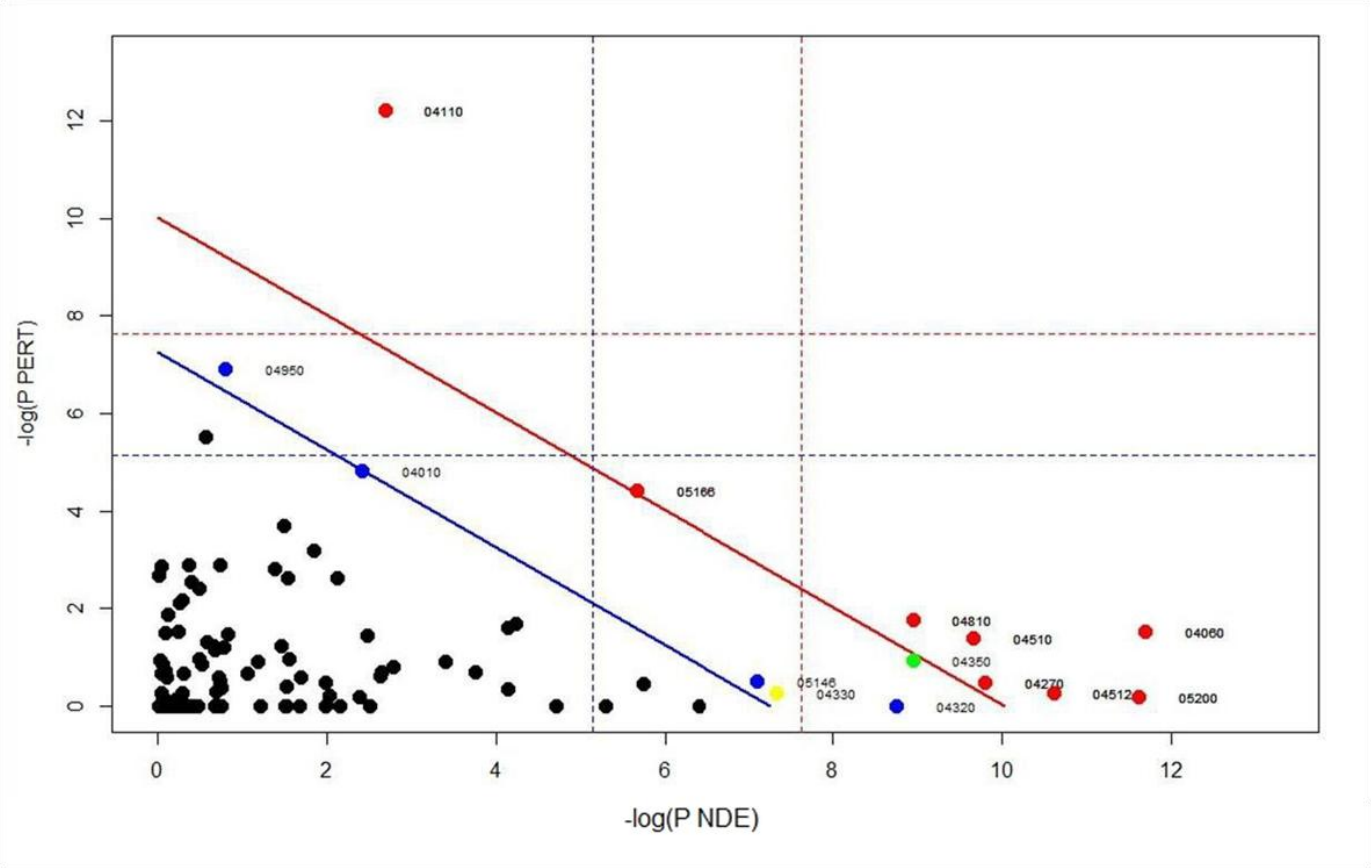

Fig. S2. SPIA analysis on up-regulated genes in AoSMCs exposed to *P. gingivalis*. X-axis indicates probability to observe differentially expressed genes on the pathway; y-axis refers to the probability to observe perturbation of genes within pathways. Each number refers to a KEGG pathway ID. Pathways above the blue line are significant at 5% after FDR correction, those above the red lines are significant at 5% after Bonferroni correction.438 up-regulated genes were analyzed by SPIA analysis. The Notch pathway is shown as a yellow dot and TGF-beta pathway is shown as a green dot.
